# Computer Assisted Assembly of Connectomes from Electron Micrographs: Application to *Caenorhabditis elegans*

**DOI:** 10.1371/journal.pone.0054050

**Published:** 2013-01-16

**Authors:** Meng Xu, Travis A. Jarrell, Yi Wang, Steven J. Cook, David H. Hall, Scott W. Emmons

**Affiliations:** 1 Department of Genetics, Albert Einstein College of Medicine, Bronx, New York, United States of America; 2 Dominick P. Purpura Department of Neuroscience, Albert Einstein College of Medicine, Bronx, New York, United States of America; Universitat Rovira i Virgili, Spain

## Abstract

A rate-limiting step in determining a connectome, the set of all synaptic connections in a nervous system, is extraction of the relevant information from serial electron micrographs. Here we introduce a software application, Elegance, that speeds acquisition of the minimal dataset necessary, allowing the discovery of new connectomes. We have used Elegance to obtain new connectivity data in the nematode worm *Caenorhabditis elegans*. We analyze the accuracy that can be obtained, which is limited by unresolvable ambiguities at some locations in electron microscopic images. Elegance is useful for reconstructing connectivity in any region of neuropil of sufficiently small size.

## Introduction

The neural circuits that create the functions of the nervous system arise from the patterns of synaptic connections between the neurons. The size of the sub-cellular structures that define synaptic contacts (synaptic densities, synaptic vesicles, and gap junctions) as well as the tiny diameter of many neurite processes (50 nm), necessitates the use of high-resolution electron microscopy of ultra-thin serial sections for a dense reconstruction. In the late 1960’s, Sydney Brenner sought to overcome the discrepancy in scale between the capabilities of electron microscopy and the typical nervous system by selecting an animal that was tiny, simple, and regular enough to allow for a complete reconstruction [1]. The efforts of the Brenner group resulted in the first complete animal connectome, the wiring diagram of the 302-neuron nervous system of the *C. elegans* adult hermaphrodite [2], [3]. This wiring diagram, fundamental to *C. elegans* research, has made possible genetic and molecular analysis of behavior in the context of known connectivity, a circumstance unique to this organism. The connectome reveals the type of every neural cell (sensory, interneuron, motor neuron) and has allowed circuit-level analysis of several worm behaviors (*e.g.* [4]–[8]).

The methodology used by the Brenner group involved marking paper prints of electron micrographs with colored pens to follow neurites through thousands of images, after which lists of synaptic contacts were written down and maps drawn by hand. The enormous, more than 12 person-year effort has inhibited further attempts at complete reconstructions in *C. elegans* or any other animals. The most labor-intensive and difficult step was analysis of the electron micrographs. In order to make determination of additional connectomes possible, we developed a PC-based software application, Elegance, to expedite this step. Using Elegance, we have determined the connectome of the posterior nervous system of the *C. elegans* adult male and repeated, for comparison, parts of the hermaphrodite connectome [9] (unpublished data available at http://wormwiring.org). The posterior nervous system of the male, which contains circuits for mating behavior, was attempted by the Brenner group in the 1970’s using their methods, but its size and complexity, due in part to branching neurons, prevented its completion [10]. With Elegance, complexity is no longer an issue. The possibility of determining a connectome from an existing electron micrographic series with a reasonable amount of effort now depends solely on the size of the dataset (number of sections, number of neuron profiles and synapses per section), the continuity of the serial stack, and the clarity of the electron micrographic images. The minimally ten-fold increase in speed of annotation and assembly we have achieved brings additional connectomes within feasible range.

### Description of Software and Database

**Figure 1:**
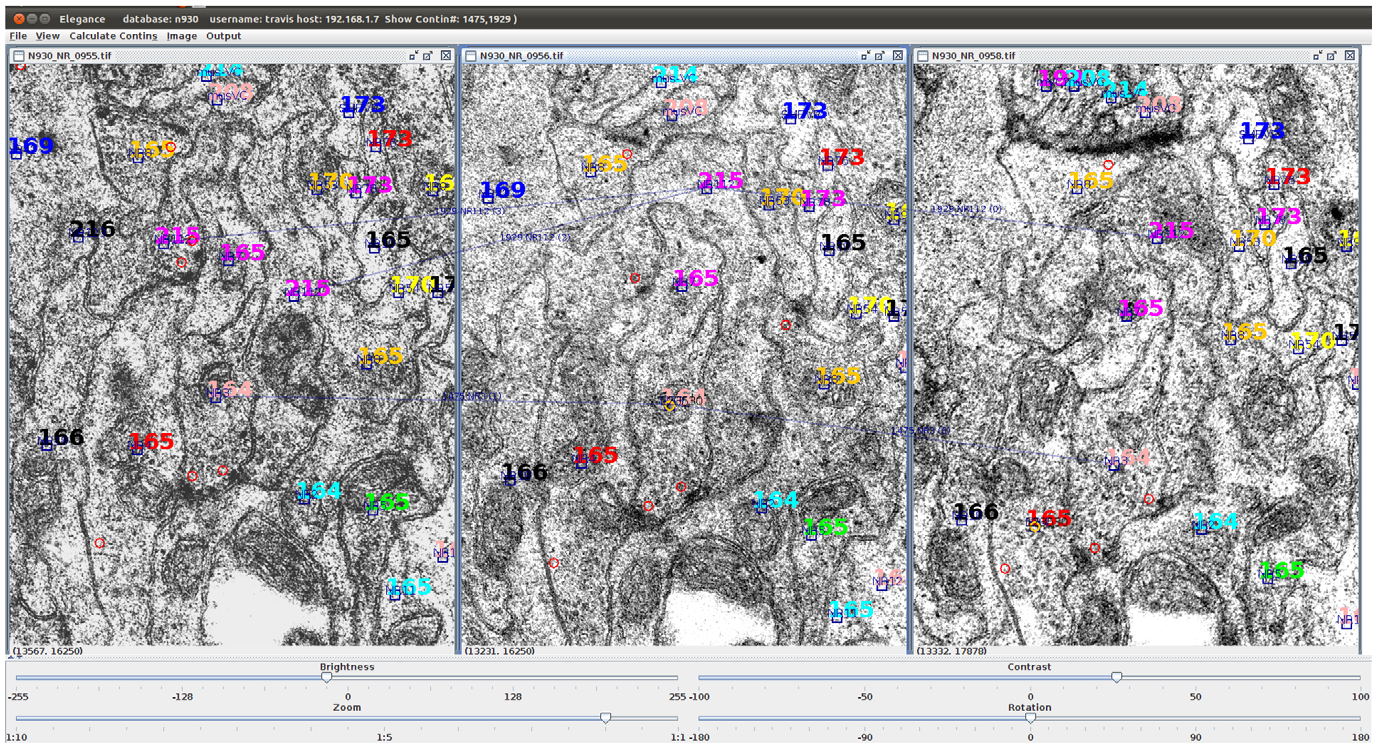
Elegance screenshot. Cell profiles are marked by open blue squares. Different chains of connected objects are labeled with a colored number for ease of recognition. The pink 215 neuron branches between the central and left-hand images. Synapses are marked by red circles.

**Figure 2:**
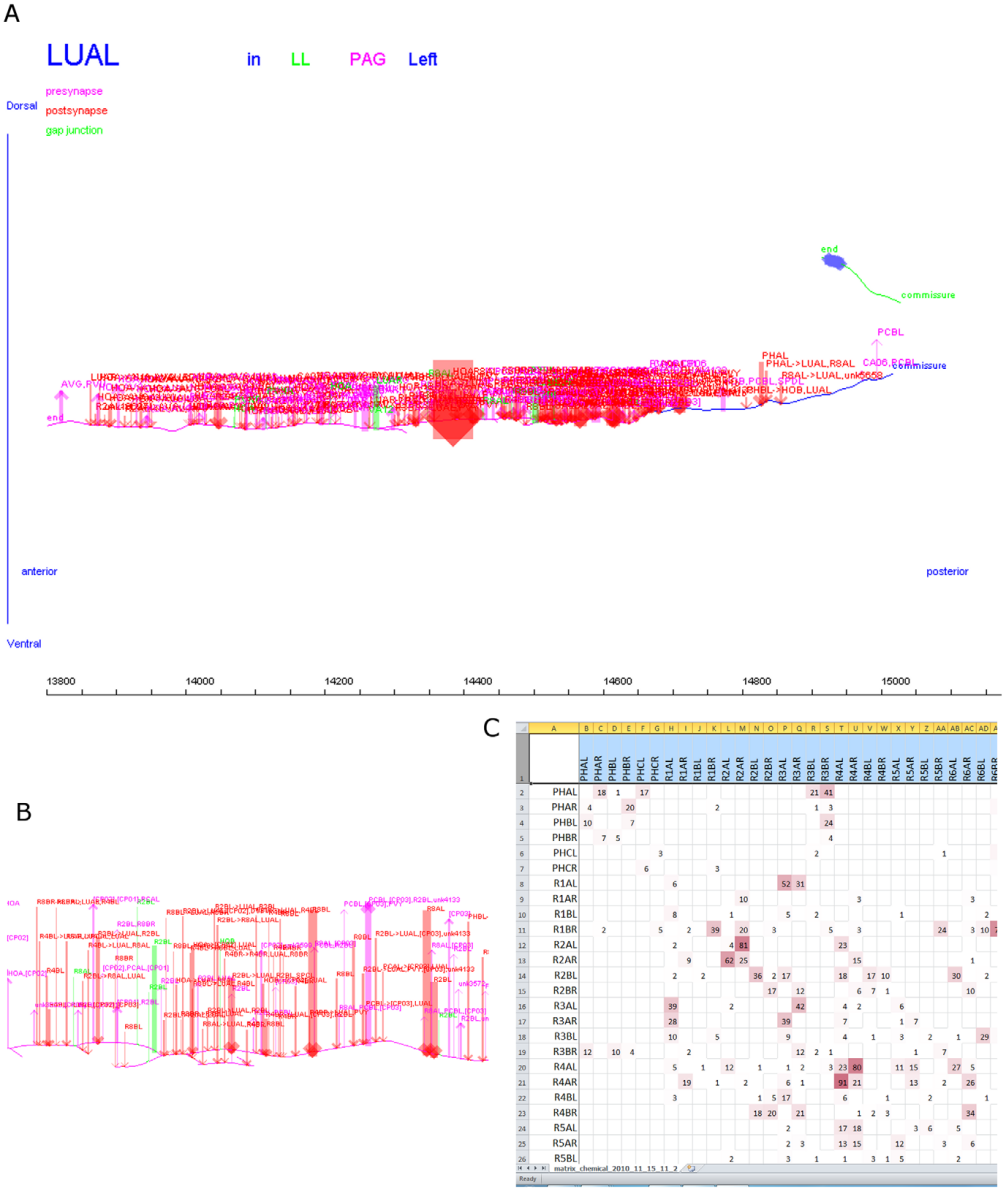
Elegance output. A. 2D map of the *C. elegans* neuron LUAL in the adult male [9]. The scale indicates section number (70–90 nm section thickness). The cell body (solid blue box) is in the left lumbar ganglion and is connected through a commissure to a process in the pre-anal ganglion which is dense with synapses. B. Enlargement of a small region of the LUAL map, showing branching architecture and locations of synapses; line thickness indicates synapse size (red: chemical input, magenta: chemical output, green: gap junctions). C. Portion of the adjacency matrix of chemical connectivity in the *C. elegans* male posterior nervous system [9], generated by Elegance and formatted in Excel. Pre-synaptic neurons to left, post-synaptic neurons at top, connection strength in number of serial sections.

Elegance is a JAVA-based program that facilitates the tracing and reconstruction of neurons and synapses across serial section images (Fig. 1). The location of a neuron profile in an image is represented by a single point and neuron reconstructions are non-volumetric skeleton diagrams. The physical location of each synapse is recorded along with synapse size. Multiple neurons can be traced simultaneously. To reconstruct a neuron, the locations of structures in images, both neurite profiles and synapses, are entered from the computer screen with the mouse. Coordinates and associated attributes are stored in a MySQL database. From the information in the database tables, Elegance draws 2D and 3D neuron diagrams and generates synapse lists and connectivity (adjacency) matrices (Fig. 2).

The MySQL database populated by Elegance contains two central tables, an Object Table and a Relationship Table. These two tables hold the essential data of the reconstruction, entered by annotating the images with the mouse. In the Object Table, each record contains a numerical identifier for an object and the object’s X, Y, Z (section number) coordinates. An object may denote the approximate centroid of a neurite profile or it may denote a presynaptic density or a gap junction.

Each record in the Relationship Table, which refers to the Object Table, holds the numerical identifiers of a pair of objects that the user wishes to link together. These may be the objects representing neurite profiles in adjacent images that are part of the same neurite. Or they may be objects identifying a single presynaptic density or gap junction that runs across multiple sections. Elegance uses the Relationship Table to assemble the elements of the connectome. Upon user request, Elegance searches the Relationship Table starting from a specified object to find all the objects linked to it in a continuous chain. For neurite objects, these chains, which are called *contins*, represent the skeleton traces of neurons. For synapse objects, contins represent individual synapses. Elegance assigns to each contin a numerical identifier. These contins, which play a central role in reconstruction using Elegance, are gradually built up as a reconstruction project proceeds until each one represents a complete neuron or a fully annotated synapse.

In both Object and Relationship Tables, along with the central data, additional fields allow the user to enter further information into the records. The identities of the pre- and postsynaptic neurites, for chemical synapse objects, or connected pairs for gap junctions, are stored in each synapse object record in the Object Table. Additional information includes user identity, date of entry, degree of certainty, membership in a contin, and so forth.

Elegance is organized to provide three main functions: 1) enter metadata about images, including section number and file locations, into an image database; 2) bring multiple adjacent serial images to the screen with overlays showing previous annotations (Fig. 1) (Elegance loads multiple adjacent images into RAM to allow fast scrolling through the stack of images with arrow keys); allow the entering of new annotations and store these in a MySQL database; 3) output selected data from the database in one of several formats (Fig. 2). Multiple projects can be handled simultaneously and multiple users can simultaneously work on a single project. Elegance is a standalone JAVA application running under Windows or Unix operating system. Implementation requires prior installation of the JAVA environment and the MySQL database. The Graphical User Interface templates are derived from ‘JFrame’ and ‘JComponent’ classes, while Windows default display features have been retained for most of the user interfaces. The Application Logic layer contains classes for functional logic, flow control and for supporting the GUI control. This layer adopts Java Advanced Imaging (JAI) API for image handling and processing to render the display and provide better GUI characteristics. It uses J-Connector for connecting to the MySQL database. TIFF and JPEG image formats are supported.

Elegance has been implemented using a Linux server (2.4 GHz Intel Core 2 Duo processors, 8 GB RAM) running the MySQL database. User terminals are similarly configured. With this configuration 4 users have simultaneously annotated images without slowing the system. The size of a reconstruction, in terms of the number of objects or number of sections involved, is unlimited. Images of 400 MB and 16,000 X 16,000 pixel dimension have been handled. The number of images that can be simultaneously loaded into RAM at each user terminal will depend on the sizes of the images and the amount of RAM. Elegance is open source. The source code, documentation for installing and running the software, and an annotated test dataset are provided as Supplemental Information available at https://wormwiring.org. Elegance is also available for download at https://github.com/Emmonslab. This laboratory is committed to maintaining the software and is prepared to offer assistance upon request.

## Results

Elegance differs from other computer packages for EM image analysis for connectomics in being designed with the sole aim of capturing as quickly as possible the minimum information necessary to obtain neuron maps and a connectivity matrix. With its simple design, a new user can become proficient with 20 min of instruction. In the first phase of reconstruction, in order to trace neurons through a stack each image must be partitioned or segmented into regions corresponding to different neurites. Some computer-assisted approaches segment the image by tracing cell boundaries. Tracing by hand, such as in widely-used Reconstruct [11] and TrakEM2 [12], is very slow, while automated approaches have yet to achieve sufficient accuracy and require correction by hand [13]–[18]. Skeleton tracing is available in TrakEM2, a more feature-rich application than Elegance, and in KNOSSOS, which does not support scoring of synapses [12], [19].

In Elegance, the image is segmented by the user placing a single point object at the center of each neurite profile. To facilitate this phase of annotation, Elegance displays three adjacent images side-by-side on the computer screen and combines in a single mouse click the operations of creating a new neurite object and connecting it to an existing neurite object in an adjacent image (Fig. 1). The user can trace very quickly by scrolling through the image stack in RAM with arrow keys. From the track of points, 2D and 3D skeleton diagrams of the neurons are drawn (Fig. 2). These neuron maps will accurately reflect the pathways of the neurites provided the image stack is well-registered.

In the second phase of reconstruction, synapses are annotated in the same way as cell profiles by single point objects. To be useful in modeling network function, the description of a connectome should include not only connectivity but also the strengths of connections. In graph theoretic terms, in which the neurons are nodes or vertices and the synaptic connections are edges, edge weight represents synaptic strength. Elegance annotation records not only the physical location of each synapse, it also allows calculation of synapse size by determining the number of physical sections the presynaptic density or gap junction traverses. Synapse size serves as a morphometric proxy for synapse functional strength [20]. Total morphological strength of synaptic interaction between each pair of cells is obtained by summing over all the synapses between them. Elegance neuron maps indicate the location along the skeleton of the synapses and synaptic partners (Fig. 2A,B). Elegance also generates lists of synapses and a connectivity matrix (weight adjacency matrix) that gives the total interaction strength for each pair of cells (Fig. 2C).

The accuracy of a nervous system reconstruction has two elements: whether the correct continuity and branching structure of the neurites has been obtained, and whether their synaptic interactions have been correctly identified. For the first of these, the ability to obtain perfect neurite architecture from an electron microscopic series is compromised only if the quality of the electron micrographs is poor, if the distance between images in any region is too great (e.g. from missing sections), or if a neurite of thin caliber close to section thickness (in the range 90–30 nm for TEM) runs in or near the plane of sectioning. Absent such difficulties, an accurate reconstruction can be expected.

Annotation of synaptic interactions has two aspects, identification of synaptic partners and estimation of synaptic strengths. The usefulness of a described connectome for quantitative functional modeling will depend on the accuracy of the weights in the weight adjacency matrix. We found in our *C. elegans* reconstructions that, in contrast to determination of neurite architecture, determination from EM images of synaptic interactions and their strengths is subject to inherent limitations that cannot be resolved unambiguously. These limitations include faint pre-synaptic densities, doubtful post synaptic partners at polyads, and uncertain gap junction structures. For this reason, different individuals annotating the same electron micrographs do not generate identical connectomes.

**Figure 3:**
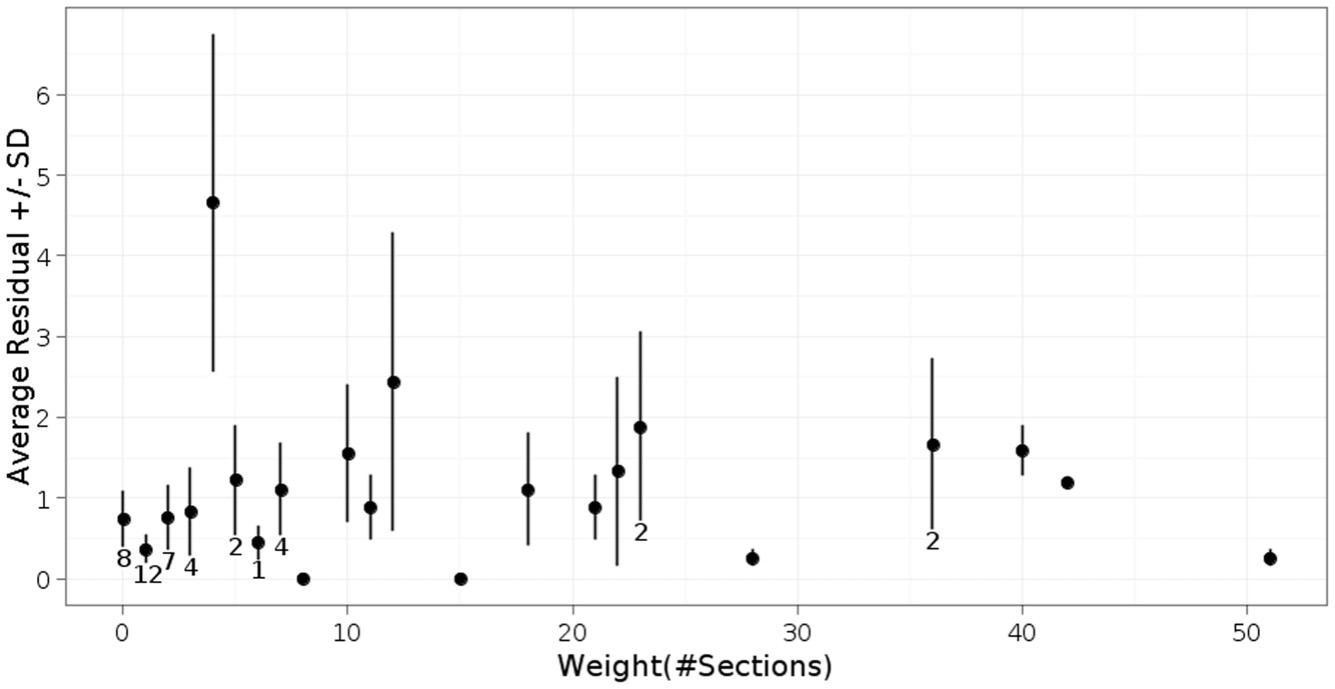
Error in determining connection strength. Average and distribution of edge weights (total amount of synaptic interaction) determined by three separate individuals for the 55 edges connecting the male-specific interneuron PVX to its 55 synaptic partners in the *C. elegans* male posterior connectome [9]. Edge weights are represented by the number of 70–90 nm serial sections of pre-synaptic densities. The weights are for a single edge except where indicated. Values of edge weights (abscissa) are the weights assigned by one of the three scorers, arbitrarily chosen. Weight values typically differed by 1 to 2 sections.

To assess the impact of these uncertainties, we compared duplicate reconstructions carried out by separate individuals. This assessment was carried out in the context of our reconstruction of the posterior connectome of the *C. elegans* male. All of the images and adjacency matrices from this study are available through our publication [9]. In a comparison of the duplicate scoring of 119 chemical synapses in the *C. elegans* male, 41% were scored identically―presynaptic density running through the same number of sections, the same post-synaptic partners. Of the remainder, 25% of the total differed in synapse size, 20% of the total differed in a post-synaptic cell, and 14% of the total were annotated by only one of the two people. To estimate how strongly these differences affected the measured weights in the adjacency matrix, we compared three independent reconstructions of a male-specific interneuron, PVX. PVX is connected to 55 neurons via 420 chemical synapses [9]. Differences between the three scores of the 55 edge weights averaged 1–2 sections, and was approximately independent of edge weight (Fig. 3). Hence the estimated percent uncertainty falls with increasing weight from around 10% for weights of 10 sections or less to 2% for edge weights of 40 sections or more. Similar values are likely to hold for scoring gap junctions.

Does this error rate compromise a useful determination of the connectome? We compared the similarity of the connectivity of the three duplicate annotations of the PVX neuron to the similarity of the connectivity of presumptively equivalent neurons in the same animal. For equivalent cells, we compared left/right homologous neurons [9]. Our duplicate annotations were significantly more similar to each other than the equivalent neuron pairs are to each other (data not shown). Therefore the error in our measured connectome is less than the differences between presumptively equivalent neurons and we can be comfortable that the differences between neurons in our reconstruction are real and not a result of measurement error.

## Discussion

Under optimal conditions―well-aligned, high quality images and neurons running mostly orthogonal to the plane of section, as for example in the *C. elegans* ventral nerve cord―data points can be entered into Elegance at a rate of 1000 per hr. We reconstructed the *C. elegans* hermaphrodite nerve ring, a more difficult region where neurons run nearly parallel to the plane of section, which required 60,000 objects, at a rate of approximately 600 per hr. These values may be used to estimate the time required for a contemplated reconstruction project in any system. The overall increased speed achieved allowed us to easily complete a new reconstruction of the longest series previously reconstructed for the hermaphrodite connectome (the N2U series, see [2]) (unpublished). This project required several months, compared to many years when carried out on paper prints by hand. Moreover, it yielded a more accurate reconstruction including synaptic weights. In a second project, a single individual reconstructed the posterior connectome of the *C. elegans* hermaphrodite from existing electron micrographs (JSE series, see [2]) in 131 hr. Reconstruction of the posterior hermaphrodite connectome had previously occupied an entire PhD thesis project [21]. These experiences lead us to estimate that Elegance provides at minimum a ten-fold increase in speed over previous methods.

Reconstructions in *C. elegans* are facilitated by the often unbranched architecture of the neurons and the relatively stable neighborhoods in which they run. Moreover, as *C. elegans* is a worm, many neurons run orthogonal to a transverse sectional plane. However, many do not, and this does not prevent their reconstruction. In certain regions, notably the pre-anal ganglion of the male, *C. elegans* material is highly similar to mammalian neuropil. Mammalian neuropil has been traced from anisotropic TEM image stacks similar to those we have used (5 nm×5 nm×50 nm) [22]. A simple modification of Elegance for scoring synapse size to accommodate the in-plane dimension can be made by allowing more than one synapse object per synapse per image.

The speed and simplicity of reconstruction with Elegance makes a more complete and accurate reconstruction possible, while providing the additional information of synapse strength. The quantitative weight adjacency matrix can be analyzed with the many tools of graph and network theory, leading to insights into network organization and function [9].

Elegance makes it possible to pursue the long-term goal envisioned by Brenner, to use *C. elegans* to identify the genetic code for a wiring diagram by analyzing presumptive connectivity mutants [1]. The post-embryonic L1 larva will require at most one tenth the number of objects as an adult, making its reconstruction feasible in one to two months once a satisfactory set of images is obtained. Comparison of the L1 connectome to those of later larval stages will reveal the process of nervous system growth and synaptogenesis, and allow investigation of whether this process is influenced by experience and learning in *C. elegans*. Studies of this type may be undertaken in any organism comparable in size to *C. elegans* or for any small region of the brain.
